# Deleting SUV39H1 in CAR‐T cells epigenetically enhances the antitumor function

**DOI:** 10.1002/mco2.552

**Published:** 2024-04-21

**Authors:** Yuning Wang, Guo Zhao, Shuhang Wang, Ning Li

**Affiliations:** ^1^ Clinical Trial Center, National Cancer Center/National Clinical Research Center for Cancer/Cancer Hospital Chinese Academy of Medical Sciences and Peking Union Medical College Beijing China

## Abstract

SUV39H1 ablation in CAR‐T cells epigenetically enhances the antitumor function (by Figdraw). (A) Schematic illustration of SUV39H1 ablation‐mediated enhanced antitumor function of CAR‐T cells. Functional CAR‐T cells eventually transformed into dysfunctional exhausted CAR‐T cells under the exposure of chronic tumor antigens, accompanied by reduced proliferation level, effector function, and stemness/memory characteristics, thereby limiting the antitumor activity so as to cause the recurrence of solid tumors. Upon genetic engineering of SUV39H1 ablation, SUV KO CAR‐T cells are endowed with increased proliferation level and stemness/memory properties, accompanied by reduced effector/exhausted phenotype. Augmented SUV KO CAR‐T cells after in vitro expansion intravenously infusion to mice achieved stronger and more persistent tumor rejection. (B) SUV39H1 ablation‐mediated epigenetic reprogramming mechanism of CAR‐T cells. Epigenetically, under the stimulation of chronic tumor antigens, exhausted CAR‐T cells were characterized by downregulation of proliferation, effector and stemness/memory‐associated genes and upregulation of exhaustion‐associated genes. SUV39H1 genetic ablation increased chromatin accessibility of stemness/memory‐associated genes and reduced chromatin accessibility of inhibitory receptors and effector‐associated genes in SUV KO CAR‐T cells, epigenetically reprogramming human T cells to express higher levels of stemness/memory genes such as KLF2, LEF1 and TCF7 and lower levels of effector/exhaustion genes.

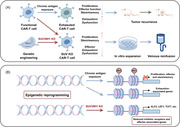

1

Recently, two back‐to‐back studies were disseminated in *Cancer Discovery*, uncovering that the genetic ablation of SUV39H1 epigenetically augmented chimeric antigen receptor‐T (CAR‐T) cell function against solid tumors.^1^, ^2^ These findings identified the critical roles of SUV39H1 in regulating T cell stemness/memory and exhaustion, which provided a promising universal translational road for SUV39H1‐based epigenetic reprogramming strategy to armor cellular therapies (Figure 1).

**FIGURE 1:**
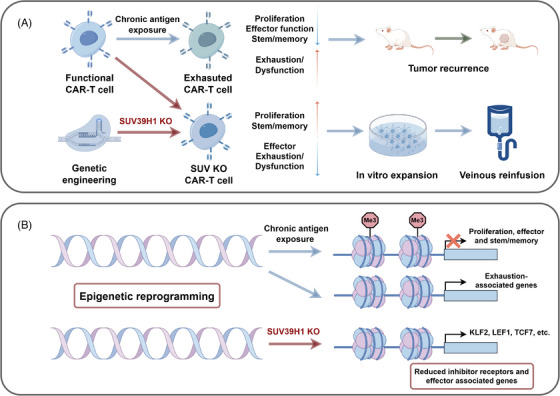
SUV39H1 ablation in CAR‐T cells epigenetically enhances the antitumor function (by Figdraw). (A) Schematic illustration of SUV39H1 ablation‐mediated enhanced antitumor function of CAR‐T cells. Functional CAR‐T cells eventually transformed into dysfunctional exhausted CAR‐T cells under the exposure of chronic tumor antigens, accompanied by reduced proliferation level, effector function, and stemness/memory characteristics, thereby limiting the antitumor activity so as to cause the recurrence of solid tumors. Upon genetic engineering of SUV39H1 ablation, SUV KO CAR‐T cells are endowed with increased proliferation level and stemness/memory properties, accompanied by reduced effector/exhausted phenotype. Augmented SUV KO CAR‐T cells after in vitro expansion intravenously infusion to mice achieved stronger and more persistent tumor rejection. (B) SUV39H1 ablation‐mediated epigenetic reprogramming mechanism of CAR‐T cells. Epigenetically, under the stimulation of chronic tumor antigens, exhausted CAR‐T cells were characterized by downregulation of proliferation, effector and stemness/memory‐associated genes and upregulation of exhaustion‐associated genes. SUV39H1 genetic ablation increased chromatin accessibility of stemness/memory‐associated genes and reduced chromatin accessibility of inhibitory receptors and effector‐associated genes in SUV KO CAR‐T cells, epigenetically reprogramming human T cells to express higher levels of stemness/memory genes such as KLF2, LEF1, and TCF7 and lower levels of effector/exhaustion genes.

In Jain's work, researchers first constructed SUV39H1‐disrupted T cell models via electroporation with Cas9 mRNA and multiple SUV39H1‐targeting guide RNAs (gRNAs).^1^ Among these candidates, the optimal editing efficiency reached ∼80%. Subsequently, they confirmed in these modified T cells without CAR structure, the elimination of the SUV39H1 gene led to the absence of SUV39H1 protein and global loss of H3K9me3 levels. Then, in order to further explore whether SUV39H1 knockout could improve the tumoricidal capacity of CAR‐T cells, SUV39H1‐disrupted, CAR‐transduced T cells and unedited CAR‐T cells were respectively infused into B cell acute lymphoblastic leukemia mouse models to compare the difference in efficacy. The experimental results displayed that SUV39H1 knockout boosted the potent tumoricidal capacity of CAR‐T cells. Specifically, nine out of 10 mice in the experimental group survived over 90 days, whereas only one out of 12 in the control group survived. Additionally, the elevated antitumor effects conferred by SUV39H1 ablation were also confirmed in PSMA‐targeted CAR‐T cell therapy against prostate cancer across three different CAR structures, indicating the therapeutic tractability and universality of this strategy. Notably, although the SUV39H1 gRNA exhibited slight off‐target activity in tests, this off‐target editing did not escalate over time and did not contribute to the improved CAR‐T cell proliferation.

Furthermore, the researchers assessed the number of CAR‐T cells in the bone marrow post‐CAR‐T cell infusion. They found SUV39H1 ablation boosted initial CAR‐T cell proliferation and reduced PD‐1 expression at day 10. Next, to further profile the influence of SUV39H1 knockout on CAR T cells, single‐cell transcriptional sequencing was conducted at three distinct time points. GSEA of differentially expressed genes showcased elevated proliferation, better memory differentiation and persistence, and diminished effector function in CAR‐T cells with disrupted SUV39H1. Further in‐depth analysis showed that these cells maintained a wider clonal repertoire. Additionally, of note, disruption of SUV39H1 resulted in a moderate suppression of cytokine secretion while enhancing the mitochondrial fitness of CAR‐T cells, in which scenario, the proliferation and cytolytic capabilities of CAR‐T cells with disrupted SUV39H1 were not compromised, even well improved at day 21.

To our knowledge, CAR‐T cell exhaustion caused by prolonged tumor antigen exposure extremely limits the tumoricidal capacity of CAR‐T cells. In this study, enhanced persistence of CAR‐T cells was observed in SUV39H1‐disrupted groups in the absence of chronic antigen stimulation, and this long‐term persistence successfully protected against repeated tumor rechallenges in the NALM6 model, also enhanced the CAR‐T cell numbers and reduced the expression of LAG3 and TIM3. Subsequently, further RNA sequencing unveiled memory‐associated transcription factors and receptors were upregulated, along with downregulation of effector/terminal‐effector state‐related transcription factors, inhibitory receptors, and genes associated with T cell exhaustion in CAR‐T cells with disrupted SUV39H1 under consecutive rechallenges, which echoed the results of paired ATACseq. Importantly, motif analysis identified the enrichment of two crucial memory‐associated transcription factors, TCF7 and LEF1, which mediated the suppression of transcriptional factors involved in effector differentiation and inhibitory receptors. The disruption of SUV39H1 maintained the expression of TCF1 and LEF1 upon repeated antigen stimulation, thereby improving the T cell memory and long‐term antitumor function.

Consistent with the above conclusions, in López‐Cobo's study, SUV39H1 serves a nonredundant role of balancing between T cell stemness/memory and effector/exhaustion phenotypes.^2^ Thus, SUV39H1‐disrupted CAR‐T cells have an improved stemness/memory and reduced effector/exhausted phenotype, thereby exerting more potent tumor rejection and more persistent protection in both lung adenocarcinoma and B‐ALL models. Single‐cell transcriptomic analysis further confirmed stem/memory cells and signatures were enriched in CAR‐T cells with disrupted SUV39H1. Consistently, CytoTRACE analysis revealed delayed differentiation properties and maintenance of stemness features in these cells. Moreover, the label transfer algorithm indicated the match between cycling CAR‐T cells and stem/memory CAR‐T cells in the lungs, elucidating enhanced self‐renewal potential in SUV39H1‐disrupted stem/memory populations. Furthermore, scATAC‐seq demonstrated SUV39H1 disruption increased chromatin accessibility of stem/memory‐associated genes in all CAR‐T cell subpopulations, including LEF1, TCF7, and so on, likewise. Noteworthy is the re‐emerging of the tumor rechallenge experiment that SUV39H1 inactivation endowed CAR‐T cells with persistent memory and enforced long‐term protection against lung adenocarcinoma and ovarian cancer rechallenges. Overall, SUV39H1 ablation imparted CAR‐T cells enhanced proliferation, stemness/memory, functional persistence, and exhaustion‐resistant properties in treating various tumors. Significantly, these works lead us to profound prospects of its potential translational values and many more opportunities.

First, CRISPR/Cas9‐based screening identified many more T cell exhaustion‐associated regulators, which were engineered in preclinical studies/clinical trials to augment CAR‐T antitumor function. DNMT3A and TET2‐mediated DNA methylation regulation and some other emerging regulators such as PRDM1, Regnase‐1, MED12, and CCNC profoundly shaped the epigenetic panorama of intrinsically modified CAR‐T cells to maintain their functional persistence.^3^ Notably, some potential risks brought by genetic knockout of epigenetic regulators were observed in previous studies such as TET2 knockout‐mediated unchecked proliferation and secondary somatic mutations, which arose us to reflect on the potential secondary tumorigenesis and severe adverse effects risks caused by uncharted off‐target effects of SUV39H1, and woke us to avoid similar events. Meanwhile, we advocate the underlying crosstalk mechanisms and multi‐targets knockout strategies should be underscored, and the resulting possible safety issues also warrant long‐term close attention.

Furthermore, the multifaceted roles of SUV39H1 in cancer cells and T cells, such as regulating tumorigenesis, progression, T cell memory formation, and exhaustion, have enlightened us to explore more possibilities. Beyond affecting the antitumor activity of T cells, SUV39H1 was previously confirmed to be overexpressed in multiple solid tumors and negatively associated with patients’ survival and immune infiltration.^4^ Thus, deleting SUV39H1 in cancer cells slowed down the tumor growth through cGAS demethylation‐mediated enhanced antitumor immunity in solid tumors.^4^ Given that SUV39H1 ablation can concurrently reverse the exhausted state of T cells and that small molecule inhibitors targeting SUV39H1 pharmacologically have equivalent effects with SUV39H1 disruption, the development of corresponding small molecule inhibitors or exploration of combination with SUV39H1‐disrupted CAR‐T cell therapy both affecting the biological behaviors of some specific cancer cells and CAR‐T cells may hold promise as a promising superposition strengthening strategy to conquer solid tumors despite of the possibility that systematic AEs exist.

Additionally, SUV39H1 was discovered to epigenetically regulate the responsiveness of CD8+ T cells to anti‐PD‐1 therapy.^5^ Genetic knockout or pharmacological inhibition of SUV39H1 in cancer cells/T cells potentiated immune checkpoint inhibitor (ICI) therapy.^4^, ^5^ So, it is rational to infer that SUV39H1‐disrupted CAR‐T cells coordinate with ICI therapy to achieve a more potent therapeutic index in clinical practice, which warrants further investigation in future work.

Altogether, these two innovative studies unveiled the mystery of SUV39H1, and first confirmed its unique role in reversing CAR‐T cell exhaustion epigenetically, thereby enhancing the antitumor function. Still, multiple challenges remain, and extensive efforts are required to facilitate its successful translation into clinics so as to benefit more cancer patients.

## AUTHOR CONTRIBUTIONS

Y. N. W and G. Z. wrote the manuscript. S. H. W. and N. L. reviewed and revised the paper. All authors have read and approved the final manuscript.

## CONFLICT OF INTEREST STATEMENT

The authors declare no conflict of interest.

## FUNDING INFORMATION

This work was supported by the grants from National Natural Science Foundation of China (82272951, 82272953), National Key Research and Development Program of China (Grant number 2023YFC2508500), Chinese Academy of Medical Science (2022‐I2M‐C&B‐B‐070) and Beijing Municipal Health Commission (BCRW20200303).

## ETHICS STATEMENT

Not applicable.

## Data Availability

Not applicable.
